# Effect of preheating on the cytotoxicity of bulk-fill composite resins

**DOI:** 10.34172/joddd.2020.003

**Published:** 2020

**Authors:** Mohammad Esmaeel Ebrahimi Chaharom, Mahmoud Bahari, Leila Safyari, Hossein Safarvand, Hajar Shafaei, Elmira Jafari Navimipour, Parnian Alizadeh Oskoee, Amir Ahmad Ajami, Mahdi Abed Kahnamouei

**Affiliations:** ^1^Department of Operative Dentistry, Faculty of Dentistry, Tabriz University of Medical Sciences, Tabriz, Iran; ^2^Dental and Periodontal Research Center, Faculty of Dentistry, Tabriz University of Medical Sciences, Tabriz, Iran; ^3^Department of Operative Dentistry, School of Dentistry, Ardabil University of Medical Sciences, Ardabil, Iran; ^4^Department of Anatomy, Faculty of Medicine, Tabriz University of Medical Sciences, Tabriz, Iran

**Keywords:** Cell survival, composite resin, tetrazolium salts

## Abstract

***Background.*** Due to the effect of pre-heating on the degree of conversion of composite resins and the possible effect on cytotoxicity, the effect of pre-heating of bulk-fill composite resins was investigated on cytotoxicity in this study.

***Methods.*** In this study, three different types of composite resin were used, including Tetric N-Ceram Bulk-Fil, Xtrafil, and Xtrabase. From each composite resin, 10 cylindrical samples (5 mm in diameter and 4 mm in height) were prepared, with five samples preheated to 68°C, and the other five samples polymerized at room temperature (25°C). Twenty-four hours after polymerization, cytotoxicity was assessed by MTT assay on human fibroblasts. Statistical analysis of data was carried out with two-way ANOVA and Sidak Post-Hoc. The significance level of the test was determined at 0.05.

***Results.*** There was no statistically significant difference between the mean percentage of cytotoxicity in terms of pre-heating (P>0.05), but the cytotoxicity of the studied composite resins was significantly different (P<0.001). The cytotoxicity of Tetric N-Ceram Bulk-fil composite resin was higher than that of the two other composite resins.

***Conclusion.*** Pre-heating of bulk-fill composite resin did not affect their cytotoxicity. In addition, the cytotoxicity of different bulk-fill composite resins was not the same.

## Introduction

Composite resins are extensively used as restorative materials in dentistry due to their esthetic, handling characteristics, and controlled working time.^1^ If the polymerization of composite resins is not adequate, the unreacted monomers remaining in the structure of composite resin might be released into the oral environment after mechanical and chemical degradation during the clinical service.^2^

Even immediately after placing composite resin restorations, monomers have been found in the saliva, dentin, and pulp.^3^ These monomers have side effects such as skin, mucous membranes, and eye irritation.^4^ In the oral cavity, lichenoid reactions have been reported around composite resin restorations.^5^ Also, Van Dijken et al^6^ reported that gingival exudate around the healthy enamel was less than that of composite resin restorations in seven days.

Pre-heating of uncured composite resins is popular among dentists as a way to improve the handling features during placement. Pre-heating of composite resins prior to light activation reduces their viscosity,^7^ and by better wetting of the cavity walls, leads to an increase in marginal adaptation^8^ and a decrease in microleakage.^9^

Furthermore, the increase in pre-polymerization temperature will result in better convergence by enhancing the mobility of monomers and radicals.^10^ Deb et al^11^ showed that increasing the temperature to 60°C before polymerization in conventional posterior composite resins significantly raises the degree of conversion. Also, improved conversion leads to better physical and mechanical properties, such as enhanced surface hardness, flexural strength, and tensile strength. Furthermore, the remaining unreacted monomers in the composite resin structure will be reduced with further conversion.^12^

It seems that a reduction in unreacted monomers can affect the cytotoxic properties of composite resins. Knežević et al^13^ found that, regardless of the chemical composition of the composite resin material, the number of living cells is affected by the pre-heating temperature.^13^ On the other hand, in another study, the effect of pre-heating on the cytotoxicity of conventional composite resin materials was not significant.^11^

During the placement of light-activated conventional composite resins, it is recommended to use increments with a thickness of <2 mm (the incremental technique). This method will create a uniform degree of conversion throughout the composite resin thickness and reduce the polymerization stress.^14^ However, the incremental technique has some disadvantages, such as wasting of time and the probability of creating voids between layers. These voids act as a weak point in the composite resin structure and will compromise its properties.^15^

Now, a new type of composite resin has been marketed, called bulk-fill, which can be cured in thicknesses of 4, 5, and 6 mm. These materials are made based on more translucent formulations, different types of resins and initiators, and new filler technologies.^1^ Nevertheless, achieving a sufficient degree of conversion at high depths in this group of composite resins is still challenging. The reason is that by increasing the depth, light efficiency is reduced due to absorption and dispersion.^16^ Unreacted monomers in the material increase by decreasing the degree of conversion at the depth of the cavity.^12^

As there is a tendency to use bulk-fill composite resins in deep cavities near the pulp, concerns have been raised about their biocompatibility with the pulp cells.^12^ Several studies have investigated the cytotoxicity of bulk-fill composite resins.^9-17^ Toh et al^18^ investigated the biocompatibility of bulk-fill composite resins with and without pre-polymerized filler in vitro. In their study, some bulk-fill composite resins with a thickness of 4 mm showed a cell viability of <70%.In another study, flowable bulk-fill composite resin showed cytotoxic effects after 24 and 72 hours.^19^

Considering that pre-heating affects the degree of conversion of composite resin monomers,^20^ and the degree of conversion might affect cytotoxicity, this study was conducted to investigate the effect of preheating on the cytotoxicity of bulk-fill composite resins. The null hypotheses of this study were as follows: I) Pre-heating does not have any effect on the cytotoxicity of bulk-fill composite resins. II) The cytotoxicity of bulk-fill composite resins will not be different from each other.

## Methods

The protocol of the study was approved by the Ethics Committee of Tabriz University of Medical Sciences (No: IR.TBZMED.VCR.REC.1397.207). Teflon cylindrical molds, with a diameter of 5 mm and a height of 4 mm, were used to prepare composite resin samples. Three types of bulk-fill composite resins, Xtrafil (XF), Xtrabase (XB), and Tetric N-Ceram Bulk-fil (TNB), were selected for the study (Table 1). Ten samples were made from each composite resin; five samples were pre-heated prior to polymerization up to 68ºC, while the other five samples were polymerized at room temperature (25ºC).

**Table 1:** Properties of the tested composite resin materials and their abbreviated codes

**Material**	**Type and color**	**Manufacturer**	**Lot No.**	**Organic matrix**	**Inorganic filler**	**Inorganic filler content percentage (W)**
**X- tra Fill (XF)**	High viscosity bulk- fill universal	Voco, Cuxhaven, Germany	1618416	Bis-GMA, UDMA, TEGDMA	*	86
**X- tra Base (XB)**	Low viscosity bulk- fill universal	Voco, Cuxhaven, Germany	1745721	Bis-EMA, UDMA, TEGDMA	*	75
**Tetric N-Ceram** **Bulk-Fill (TNB)**	High viscosity bulk- fill IVA(universal)	Ivoclar-vivadent, Schaan, Liechtenstein	W30705	Bis-GMA, UDMA, Bis-EMA	Barium glass, ytterbium trifluoride, mixed oxide	61

*Filler composition has not been specified by the manufacturer.

Accordingly, six groups were examined in terms of the type of composite resin and pre-heating (n=5) with a total number of 30 composite resin samples. Pre-heating of composite resins was carried out using a hot water bath with thermostatic control (TELEDYNE HANAU, Buffalo, NY, USA) up to 68°C. Furthermore, the material temperature was measured using a digital temperature microprobe (GBC KDM 350, KON ELCO SpA, Milano, Italy).^21^

After measuring the temperature of the composite resin, the Teflon mold was completely filled in one step with a single bulk of composite resin with a transparent strip placed on the superior and inferior parts of the cylinders to prevent the formation of the oxygen-inhibited layer. It was then cured by a light-curing device (LITEX 695C Cordless LED Curing Light, Dentamerica, USA) at an intensity of 1100 mW/cm^2^ for 20 seconds.

A calibrated radiometer (Bisco, IL, USA) was used to ensure the intensity of radiation in each use of the light-curing unit. To simulate the clinical condition, the device was placed in direct contact only with one side of the cylinder.^22^ The light-cured composite resin samples were sterilized through swabbing with 70% ethanol alcohol. Then, the samples were immersed in DMEM high-glucose culture medium (Dulbecco's Modified Eagle's Medium, Sigma Culture Chemical Co., St. Louis, Mo, USA) with penicillin-streptomycin supplements and fetal bovine serum (10% FBS [Gibco, UK] and 1% P/S [Gibco, UK]) and incubated at 37°C, a relative humidity of 95%, and 5% CO_2_ for 24 hours. The original solution of the extract of composite resin samples was the same as the culture medium in which the samples had been immersed.^18^ For the evaluation of the MTT, the original extract solution was diluted with the addition of the fresh medium as follows:^23^

100%: One part of the original extract

75%: Three parts of the original extract + one part of the fresh medium

50%: One part of the original extract + one part of the fresh medium

25%: One part of the original extract + three parts of the fresh medium

A cell line of human fibroblasts was obtained from the National Cell Bank of Iran (NCBI, Pasteur Institute of Iran). The cells with a density of 2×10^4^ cells/well were incubated in 96-well plates at 37°C and 5% CO_2_ for 24 hours.^18^ Each dilution was examined in six wells of plates. In each test, six wells containing human fibroblast cells without composite resin extraction were used as the control group. Finally, the cell vitality was determined by the MTT assay.

MTT dye (3-4.5-dimethylthiazol-2-yl-2, 5-diphenyltetrazolium bromide) (Sigma, USA) was dissolved in a solution of PBS (phosphate-buffered saline) (Gibco, UK) at a concentration of 5 mg/mL. Then, 10 mL of the MTT solution was added to each well containing 100 mL of culture medium. The yellow MTT solution was converted to an insoluble purple formazan over 5-hour incubation with mitochondrial dehydrogenase enzyme. Finally, the medium on the cells was completely removed and replaced with 200 μL of DMSO (dimethyl sulfoxide) (Merck, German) to dissolve the formazan produced by the cells. The optical density of the formazan solution was determined using an ELISA reader (Organon Teknika, Netherlands) at a wavelength of 540 nm, and the percentage of cytotoxicity was calculated as follows:^23^

Cytotoxicity percentage = 1 – the mean absorbance of toxicant-treated cells/mean absorbance of control ×100

### Statistical analysis

At each of the culture medium concentrations (100%, 75%, 50%, and 25%) separately, two-way ANOVA was used to evaluate the effect of pre-heating on the cytotoxicity in terms of the composite resin type. The utilized post hoc test was Sidak. Data were analyzed using SPSS 17, and the significance level of the test was determined at 0.05.

## Results

**Figure 1:**
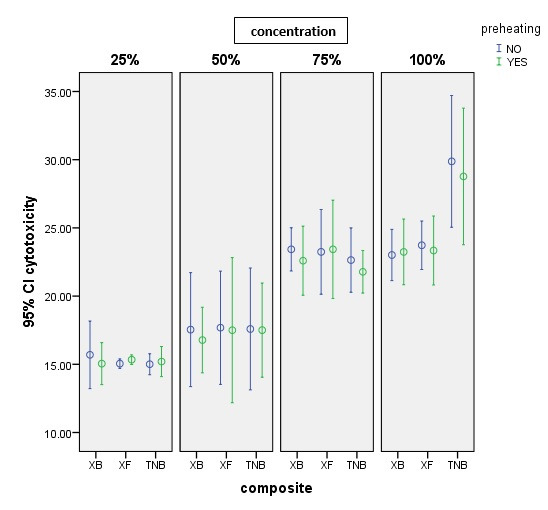
Error-bar cytotoxicity diagram in terms of the type of composite resin and the pre-heating variable.

Table 2 shows the means and standard deviations of cytotoxicity percentages in terms of pre-heating and the type of composite resin (in the cell culture medium with the studied concentrations). Also, the error-bar diagram is presented in Figure 1.

**Table 2:** Means and standard deviations of cytotoxicity, in terms of pre-heating and type of composite resin (in cellular culture medium with different concentrations)

		**Concentration**							
**Composite type**	**Preheating**	**100%**		**75%**		**50%**		**25%**	
		**SD**	**Mean**	**SD**	**Mean**	**SD**	**Mean**	**SD**	**Mean**
**XB**	No	1.51	23.01	.630	23.42	3.36	17.54	1.99	15.69
	Yes	1.94	23.23	2.04	22.59	1.93	16.77	1.23	15.04
**XF**	No	1.43	23.72	2.50	23.24	3.34	17.68	0.21	15.04
	Yes	1.58	23.34	2.90	23.43	4.28	17.49	0.22	15.34
**TNB**	No	3.88	29.87	1.89	22.63	3.59	17.58	.610	15.00
	Yes	4.03	28.77	1.25	21.77	2.78	17.50	0.88	15.20

Two-way ANOVA for each concentration showed no statistically significant difference between the mean cytotoxicity percentages in terms of pre-heating at any of the four concentrations (P>0.05). At a concentration of 100%, there was a significant difference between the mean percentages of cytotoxicity in the three types of studied composite resins (P<0.001). However, at three concentrations of 25%, 50%, and 75%, this difference was not statistically significant (P>0.05). There was not a significant interaction between pre-heating and composite resin type at any of the four concentrations (P>0.05).

At a concentration of 100%, the results of post hoc Sidak test (α=0.05) revealed a statistically significant difference between the mean cytotoxicity percentage of the TNB composite resin and its mean in both XB and XF composite resins; the mean percentage of cytotoxicity in TNB composite resin was higher than that of the two other composite resins (P<0.001). However, there was no statistically significant difference between the mean cytotoxicity percentages of XF and XB composite resins (P>0.05).

## Discussion

In this study, the cytotoxic effects of pre-heated and non-pre-heated bulk-fill composite resins were investigated in a human fibroblast cell line. In general, cell culture is used to evaluate the cytotoxicity potential of dental composite resins. There are several in vitro test models for the evaluation of cytotoxicity of dental biomaterials. They include the direct contact method, where the materials directly contact the cellular layer, the indirect contact method, where a barrier is placed between the cell and the biomaterial layer, and the extract method, where the extracts of the materials are placed in contact with cells. The best in vitro test is a test that can better simulate in vivo conditions. In a study by Lim et al,^24^ these three test methods for composite resin materials were compared, and the results indicated significant and strong correlations between all the three methods. Also, the extract test was more sensitive than contact tests. After the test model, the next factor is cell type selection. Studies have employed different techniques and primary or permanent cells.^25,26^ Most researchers prefer permanent cell lines due to their morphology and homogeneous growth characteristics. The reason is that primary cells are taken from different donors, which will differ in their developmental and cultural characteristics. Nevertheless, permanent cell lines have a poor simulation of the oral environment compared with primary cells.^27^

Previous studies have reported that the pre-heating of composite resins can enhance the rate of polymerization and the degree of conversion, and reduce the amount of uncured monomers by temporarily reducing the viscosity.^28,29^ However, Marigo et al^30^ showed that different degrees of conversion of composite resins do not necessarily lead to different cytotoxic effects. Therefore, pre-heating does not change the degree of conversion of composite resin to the extent that it affects its cytotoxicity.

The results of this study also indicated that pre-heating of the bulk-fill composite resins used did not have a statistically significant effect on their cytotoxicity, and the study was unable to reject the first hypothesis. Thus, the pre-heating of the composite resins did not cause adverse reactions, and the cytocompatibility of the components released from the composite resin was not affected. This result was consistent with those of a study by Deb et al.^11^ In their reports, the pre-heating of composite resins did not significantly influence their cytotoxicity. However, the study by Knežević et al^13^ suggested that cell viability was affected by the pre-heating temperature.

In their study, the highest cell viability was reported in composite resins pre-heated up to 54°C with 40 seconds of curing time and at 37°C with 20 seconds of curing time. They used lymphocytes to investigate cell viability by direct contact with composite resin samples (not their extracts), and staining with different techniques and materials. However, in the mentioned study, similar to ours, there was no statistically significant difference at 68°C.^13^

The results in the present study also indicated that different composite resins had different cytotoxicity levels. Therefore, the second hypothesis was rejected. This result was consistent with the results of other studies.^11,19,31^ The cytotoxicity of TNB composite resin was higher than that of XB and XF composite resins at 100% concentration, and no statistically significant difference was observed between XF and XB composite resins.

In a study by Nascimento et al,^31^ the results of cytotoxicity with the MTT assay were similar for XB and XF composite resins. Kamalak et al^19^ found different cytotoxic results for bulk-fill composite resins. The differences in the cytotoxicity of XB and XF composite resins with TNB composite resins seem to be related to their chemical composition.

For example, a higher filler percentage might reduce the solubility of the composite resin. There are many reports suggesting that composite resins with a higher filler content offer a lower solvent adsorption rate compared to composite resins with low filler content.^32,33^ In addition, lower solvent adsorption might result in the lower release of components.^34^ According to the manufacturer’s information, the filler percentages of TNB bulk-fill composite resins, XF, and XB are 61%, 86%, and 75%, respectively.

Therefore, the higher cytotoxicity of the TNB is probably related to a lower filler content of this composite resin and higher release of components compared to other composite resins. On the other hand, TEGDMA monomer has a synergistic effect on the extent of polymerization in the composite resin structure.^34^ According to the manufacturer’s information, there is no TEGDMA monomer in the chemical structure of TNB composite resins. This molecule is present in the structure of XB and XF composite resins and might cause enhanced polymerization in them and reduce their monomer elution compared to TNB.

Furthermore, the TNB composite resin contains pre-polymerized fillers. Unreacted residual or pendant double bands of C-C in these fillers might increase the leachable monomers.^35^ In addition to the leaching of unreacted monomers, the release of initiators, other organic matrix additives, and metallic ions from mineral fillers might also cause cytotoxicity of the composite material. Fluoride in the chemical structure of some composite resins can be cytotoxic for tissues through several mechanisms, such as inhibiting the activity of enzymes, producing reactive oxygen species, destroying the antioxidant defense system, as well as inducing inflammation and apoptosis.^18^ Among the studied composites, TNB composite resin had fluoride in its chemical composition, and this ion might lead to the more significant cytotoxic results for this composite resin.

In a study by Marigo et al,^30^ there was no statistically significant difference in the cytotoxicity of composite resins. In their study, the effect of cytotoxicity of flowable composite resin was investigated on human pulp cells. The utilized cells were not cell lines, where the dimensions of the examined samples were smaller than those of our study samples. Also, Rodríguez-Lozano et al^17^ examined the effects of cytotoxicity of two flowable bulk-fill composite resins on pulp and PDL stem cells. They found no significant difference between the two composite resins. The cells employed in that study were not cell lines and were extracted from the impacted third molars of the patients. Additionally, the composite resins studied by them were different from those of the present study.

In this study, only a cell line was used, which was a limitation of the study. These cells had a weaker clinical simulation condition. Also, in the clinical use of dental materials, there are barriers, such as dentin. In future studies, a combination of different curing methods, other cell lines, or primary cells, comparison of the direct and indirect contact methods, and greater clinical simulation conditions can be addressed. Recently, in vivo and in vitro studies have indicated that monomers have interactions with the immune system, genotoxicity, estrogenicity,^36^ hypersensitivity, and the production of active oxygen species.^19^ These parameters can also be examined to gain deeper insights in this regard.

## Conclusion

This study suggested that pre-heating of bulk-fill composite resins up to 68°C did not affect the toxicity of human fibroblasts. Furthermore, the cytotoxicity of different bulk-fill composite resins varied depending on their chemical composition. Nevertheless, future studies are required to further investigate the cytotoxicity of these materials.

## Authors’ Contributions

The concept and the design of the study were developed by MEEC, HS, EJN, and LS. The MTT method was carried out by HSH. Data entry and statistical analyses were carried out by MB and AAA. The manuscript was written by HS, LS, PAO, and MAK. All the authors participated in the literature review. All authors have read and approved the final manuscript.

## Acknowledgments

The authors would like to thank the Dental and Periodontal Research Center of Tabriz University of Medical Sciences.

## Funding

This research was carried out by financial support of the Dental and Periodontal Research Center, Tabriz University of Medical Sciences.

## Competing Interests

The authors declare no competing interests with regards to the authorship and/or publication of this article.

## Ethics approval

This research was approved by Research Ethics Committee of Tabriz University of Medical Sciences in 2018.
